# Antioxidant, Antibacterial and Antischistosomal Activities of Extracts from *Grateloupia livida* (Harv). Yamada

**DOI:** 10.1371/journal.pone.0080413

**Published:** 2013-11-29

**Authors:** Zebin Jiang, Yicun Chen, Fen Yao, Weizhou Chen, Shuping Zhong, Fuchun Zheng, Ganggang Shi

**Affiliations:** 1 Department of Pharmacy, First Affiliated Hospital, Shantou University Medical College, Shantou, China; 2 Department of Pharmacology, Shantou University Medical College, Shantou, China; 3 Department of Cardiovascular Diseases, First Affiliated Hospital, Shantou University Medical College, Shantou, China; 4 Marine Biology Institute, Shantou University, Shantou, China; 5 Department of Biochemistry and Molecular Biology, Keck School of Medicine, University of Southern California, Los Angeles, California, United States of America; Rochester Institute of Technology, United States of America

## Abstract

The present study was designated to evaluate the antioxidant, antibacterial and antischistosomal activities of Grateloupia livida (GL) extracts *in vitro*. A GL Ethanol extract (EE) was separated into petroleum ether (PE), ethyl acetate (EA), n-butyl alcohol (BuOH) and aqueous (AQ) fractions to fractionate the polar and non-polar compounds in the EE. Extracts antioxidant activities were evaluated in vitro by DPPH radical-scavenging, deoxyribose radical scavenging, and β-carotene bleaching assays, all using butylated hydroxytoluene (BHT) as the reference antioxidant compound. The most effective antioxidant properties were observed in the PE fraction in all three assays. Antimicrobial testing showed that the PE fraction exhibited broad-spectrum antimicrobial activity, with the PE fraction also exhibiting strong activity against the human pathogenic trematode S. *japonicum* adult worm. In order to investigate the relationships between bioactivity and chemical composition, the chemical composition of the PE fraction was analyzed by gas chromatography-mass spectrometry (GC-MS). In total, 25 components were identified in the PE fraction, most of which have known antioxidant and antimicrobial activities. However, none of the compounds have reported activity against Schistosoma, suggesting that the schistosomicidal activity of the PE fraction may be related to minor constituents present in the extract, or governed by more intricate synergistic or additive relationships. Finally, fractions with the greatest biological activity displayed neither cellular cytotoxicity, at concentrations up to 100 ug/ml, or acute oral toxicity in mice, at doses up to 2000 mg/kg. Based on antioxidant, antimicrobial, antischistosomal activities, and low toxicity, the PE fraction possesses properties useful for food preservation and overall improvement of human health.

## Introduction

Reactive oxygen species (ROS) such as the hydroxyl radical, superoxide anion and hydrogen peroxide radicals are formed in human cells through endogenous metabolism and result in extensive oxidative damage that in turn leads to geriatric degenerative disorders, cancer, and a wide range of other human diseases. [1] Antioxidants are effective in protecting living organisms against ROS-mediated oxidative damage, and several synthetic antioxidants are commercially available, such as butylated hydroxyanisole (BHA), butylated hydroxytoluene (BHT) and propyl gallate (PG). [2] However, due to safety issues and consumer demand, there has been considerable interest in replacing synthetic antioxidants with natural plant-based alternatives. [3] Several studies report a positive correlation between increased dietary intake of natural antioxidants and reduced coronary heart disease, reduced cancer mortality and longer life expectancy. [4], [5]

Marine algae have attracted attention in the search for natural bioactive compounds that may be used for new medicinal and functional food ingredients. Approximately 8,000 species of marine algae have been identified and grouped into different classes, including brown, red, and green seaweeds,[6] which have enormous potential to be sources for antioxidant, antimicrobial, antiviral, and antitumor drugs.[7]

Grateloupia livida (Harv) Yamada (GL), a red seaweed belonging to Rhodophyta, Rhodophyceae, Gigartinales, Halymeniaceae, Grateloupia,[8] is mainly distributed in the South China Sea. [9] Local people use GL as febrifuge, antidiarrhoeic, antibacterial, and anthelmintic agents for the treatment of ascariasis and seatworm infections, sore throat, stomachache and dysentery. Many studies of the Grateloupia family demonstrate biological activity, including antioxidant, anticholinesterase and antityrosinase activities in Grateloupia lancifolia extracts, [10] anti-HIV-1 activity of polysaccharides from Grateloupia longifolia and Grateloupia filicina, [11] antioxidant activity of Grateloupia filicina extracts, [12] antioxidant enzymatic activities in Grateloupia turuturu. [13]

Despite extensive research on the bioactive potential of extracts from the Grateloupia family, few studies have characterized the bioactive activities of GL, which is ubiquitous, easily cultivated and an important natural resources of the locality. Hence, in the present study, we demonstrate potent antioxidant, antibacterial and antischistosomal activity of GL extracts, using various in vitro assays, and characterize the chemical composition of active fractions by gas chromatography-mass spectrometry (GC-MS). Furthermore, the toxicity of active fractions was also tested with the aim of identifying novel nutraceuticals to be further explored as potential functional foods or nutraceuticals.

## Materials and Methods

### Plant Materials

Grateloupia livida (Harv). Yamada was collected at Nan Ao Island, Shantou Guangdong Province, PR China, and identified by the Nan Ao Marine Biological Research Station of Shantou University in Guangdong Province. The GL was washed thoroughly with deionized water and dried in the shade at 30°C for 24 h. The dried seaweed was then powdered and stored at −20°C until use.

The milled sample (50 g) was extracted twice with 95% ethanol (500 mL) at 70°C for 3 h. The crude extract was concentrated using a rotary evaporator and a vacuum drier at 30°C, then dissolved in distilled water and partitioned sequentially in three different solvents, petroleum ether (PE), ether ethyl acetate (EA), n-butyl alcohol (BuOH) and an aqueous fraction (AQ), to fractionate the polar and non-polar compounds in the crude extract. The resulting solvent fractions were concentrated by rotary evaporation and dried in a vacuum oven at 30°C, and the aqueous fraction (AQ) was concentrated by rotary evaporation and freeze-dried. The crude extract and its solvent fractions were stored in the dark at 20°C before analysis.

### Chemicals and Reagents

Alkane C8–C20 (mixture No. 04070) and C21–C40 (mixture No. 04071) standard solutions were from Fluka Chemika (Buchs, Switzerland). 2,2-Diphenyl-1-picrylhydazyl (DPPH), β-carotene, butylated hydroxytoluene(BHT), ascorbic acid and linoleic acid were purchased from Sigma-Aldrich Chemical Co. (St. Louis, MO, USA). 2-Deoxy-D-ribose was purchased from Amresco. 2-Thiobarbituric acid and EDTA were obtained from Aladdin (Shanghai, China). All other chemicals and solvents (e.g. ethanol, H_2_O_2_, Tween-40) were of analytical grade. Ultrapure water was used for the experiments.

### Antioxidant property assays of extracts

#### DPPH radical-scavenging system

The DPPH radical scavenging capacity of each herbal extract was evaluated according to Blois, with minor modifications.[14] DPPH radical was prepared in ethanol to a final concentration of 5×10^−4^ mol/L. Different amounts of samples at a concentration of 10 mg/mL (solid extract dissolved in 70% ethanol), was added to 50 µl of freshly prepared DPPH radical solution. Finally 70% ethanol was added to a final volume of 250 µl, and the mixture was kept in the dark for 30 min. The absorbance of the reaction mixture was measured at 517 nm. A control was measured in the same way except that the extract was replaced by 70% ethanol. All experiments were carried out in triplicate. The scavenging activity was calculated by the equation:
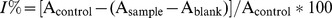



where A_sample_ is the absorbance of the sample and A_control_ is the absorbance of the control. BHT was used as positive control.

#### Deoxyribose radical scavenging activity

Deoxyribose non-site-specific hydroxyl radical scavenging activity of the ethanol extract and fractions was determined according to the method of Gutteridge (1987) with minor modifications[15]. Briefly, 1 mL of the reaction solution consisted of different volumes of sample (10 mg/ml), 0.1 ml 1 mM FeCl_3_, 0.1 ml 1.04 mM EDTA, 0.1 ml 20 mM H_2_O_2_, 0.1 ml 2 mM L-ascorbic acid and 0.1 ml 60 mM deoxyribose in potassium phosphate buffer (pH 7.4 50 mM). Then, the reaction mixture was incubated for 1 h at 37°C, after addition of 1 mL of 2.8% (w/v) trichloroacetic acid and 1 mL of 1% (w/w) thiobarbituric acid (1% in 50 mM NaOH). Deoxyribose degradation was measured by the thiobarbituric acid reaction. The reaction was further heated in a boiling water-bath for 15 min and absorbance was measured at 532 nm against a blank. All experiments were carried out in triplicate.
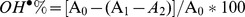



where A_0_, A_1_, A_2_ represent the absorbance of control, samples, blank, respectively. BHT was used as the positive control.

#### Beta-carotene bleaching (BCB) assay

Antioxidant activity in the musts was assessed with the β-carotene/linoleate model system[16]. For this purpose, a solution of β-carotene was prepared by dissolving 2 mg of the compound in 10 mL of chloroform. Linoleic acid (0.02 mL) and Tween 40 (0.2 mL) were subsequently added, and the mixture was left standing at 20°C for15 min. After evaporation of the chloroform in a rotary evaporator at 40°C, 50 mL of oxygen-saturated distilled water at 25°C was added and the mixture was vortexed vigorously (1 min) to form an emulsion (β-carotene/linoleic acid emulsion). Different volumes of sample and 100 µL of the emulsion per well were added to each well of a 96-well microtiter plate. The micro plate was placed on a horizontal shaker and shaken at 100 rpm (for 1 min). A control sample was also prepared in parallel. Absorbance measurements (470 nm) were made at t = 0 min and after incubation at 50°C for 120 min. All experiments were carried out in triplicate. Antioxidant activity was expressed as the percent of inhibition with respect to the control sample and calculated as follows:
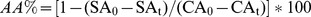



where SA_0_ and CA_0_ are the absorbance values of the sample and the control determined at 0 min; the SAt and CAt were the absorbance values of test sample and control measured after 120 min. BHT was used as the positive control.

### Antimicrobial susceptibility testing and determination of minimum inhibitory concentration (MIC)

A broth microdilution method was used to determine the MIC against susceptible microorganisms using the KB method according to the National Committee for Clinical Laboratory Standards. Three strains, *Staphylococcus aureus ATCC* 25923, *Escherichia coli ATCC* 25922 and *Pseudomonas aeruginosa ATCC* 27853 were ATCC (American Type Culture Collection) standard bacteria; the other three strains, *S aureus* 2500, *E coli* 3529 and *P aeruginosa* 2470 were isolated from the clinic. Briefly, a suspension of the tested microorganism (0.1 L of 10^8^ cells/mL) was spread on solid media plates. A serial 2-fold dilution of GL extract from 0.5–8 mg/ml was prepared in 50 µL Mueller Hinton broth (MHB) in each well of a 96-well microtiter plate. Freshly grown microbial suspensions in MHB were standardized to a cell density of 1.5×10^8^ (McFarland No. 0.5) and added to the wells (50 µL). After incubation at 37°C for 24 h, the MIC was defined as the lowest concentration of GL extract at which the microorganism did not demonstrate visible growth.

### Parasite preparation

S. *japonicum* cercariae (strain isolated in Jiangsu, China), hatched from infected *Oncomelania hupensis*, was provided by the Department of Snail Biology, Jiangsu Institute of Parasitic Diseases. Mice (C57BL/6), weighing 22∼24 g, were purchased from the Shanghai Sub-Center of Experimental Animals, Chinese Academy of Sciences, and raised in the Department of Experimental Animals, Jiangsu Institute of Parasitic Diseases.

Each mouse was infected with 50 S. *japonicum* cercariae by abdominal skin penetration. All mice were sacrificed on day 45 post-infection, and S. *japonicum* adult worms were collected by portal vein perfusion. [17]

### In vitro activity against adult Schistosoma japonicum worms

S. *japonicum* worms obtained from mice (C57BL/6, male, 22∼24 g, each infected with 50 cercariae) were washed in RPMI 1640 medium, kept at pH 7.5 with HEPES 20 mM and supplemented with penicillin (100 UI·mL^−1^), streptomycin (100 mg·Ml^−1^) and 10% bovine fetal serum (Gibco) [18], [19]. After washing, two pairs of adult worms were transferred to each well of a 24-well culture plate containing 2 mL of the same medium. The worms were cultured for 30 to 60 min at 37°C in a humidified atmosphere containing 5% CO_2_, and then different concentrations of extracts (25, 50 and 100 ug/ml) dissolved with 1% DMSO in RPMI 1640 medium were added. Control worms were treated with equal volumes of RPMI 1640 or DMSO, and worms treated with 30 ug/mL PZQ were also observed. Parasite survival was monitored under an inverted microscope (Leica, Germany) at 24, 48 and 72 h. Parasite death was defined as having no motor activity during 2 min of continuous observation as well as morphological and tegumental alterations [20]. This part of the experiment was completed in the Shanghai Sub-Center of Experimental Animals, Chinese Academy of Sciences.

All experiments were performed in quadruplicate using RPMI 1640 medium and RPMI 1640 with 1% DMSO as negative control groups and 30 ug/ml PQZ as positive control group.

### Gas chromatography-mass spectrometry analysis

GC-MS analysis involved an Agilent 7890 GC equipped with a quadrupole 5975 mass spectrometer(Agilent Technologies, CA, USA) and an HP-5MS 5% phenyl methyl Silox-bonded phase column (i.d., 0.25 mm; length, 30 m; film thickness, 0.25 um). Oven temperature was maintained at 80°C for 3 min initially and then raised to 160°C at a rate of 8°C/min; followed by raising to 216°C at a rate of 2°C/min, then raising to 280°C at a rate of 4°C/min and finally held at 280°C for 13 min. The MS conditions were: ionization voltage, 70 eV; emission current, 10 mAmp; scan rate, 1 scan/s; mass range, 45–450 M/Z; trap temperature, 150°C, and transfer line temperature, 280°C. Operating conditions were: injector temperature, 280°C; FID temperature, 280°C, and carrier (He) flow rate, 0.8 ml/min. Samples were injected splitless. 2 ul sample dissolved in ethyl acetate was injected.

Identification of the constituents was based on comparison of their Kovats Index (KI) and the MS fragmentation pattern with reference composition in the database of the NIST Mass Spectral Search Program (NIST 08 mass spectral database, National Institute of Standards and Technology, Washington, DC, USA). To determine the KI value of the components, a commercial aliphatic hydrocarbon mixture (Sigma-Aldrich) was added to the essential oil before injection it into the GC/MS and analyzed under the same conditions as above. The following quasi-linear equation for the Temperature Programmed Kovats Index was:
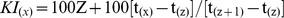



where KI(x) is the temperature-programmed Kovats Index of interest and t_n_, t_n+1_, and t_x_ are the retention times in minutes of the 2 standard n-alkanes containing n and n+1 carbons, and index of interest, respectively.

### Analysis of toxicity

#### Cell culture

The RAW264.7 murine macrophage cell line was ordered from the American Type Culture Collection (ATCC, TIB-71). Cells were maintained in complete medium containing DMEM, 10% fetal bovine serum (FBS), penicillin (100 U/mL) and streptomycin (100 µg/mL) in a humidified incubator with an atmosphere of 95% air, 5% CO2 at 37°C.

#### Determination of cytotoxicity

We used an MTT assay to measure cell death occurring in the cultures, via measurements of the formation of dark blue formazan dye crystals resulting from the reduction of the tetrazolium ring of MTT. [21] The reduction of MTT is believed to occur primarily in the mitochondria via the activity of succinate dehydrogenase, therefore providing a measure of mitochondrial function. After incubation of 1*10^6^ RAW264.7 cells in medium supplemented with GL extracts or distilled water for 24 h, the cells were treated with the MTT reagent for 6 h, until purple precipitates formed. These precipitates were then dissolved with a detergent reagent, after which we measured the absorbance at a wavelength of 595 nm, in order to determine the degree of MTT reduction which is proportional to the quantity of live cells in the culture. All experiments were performed in triplicate, and repeated twice. The number of living cell was calculated using the following equation.
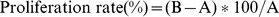



where A is the optical density without sample, and B is the optical density with sample.

### Acute Oral Toxicity – Fixed Dose Procedure. Fixed Dose

Animal Selection. Kunming (KM) mice weighing 22–25 g were maintained by Animal Center of Southern Medical University (Guangzhou, China). All animal work was performed according to the international animal welfare guidelines, and protocols were approved by Shantou University Medical College Institutional Animal Care and Use Committee.

Procedure was adopted to evaluate the acute oral toxicity of GL extracts and solvent-partitioned fractions according to the Organization for Economic Co-operation and Development (OECD) Guidelines for the Testing of Chemicals. [22] Female mice were selected for this experiment, because females are generally slightly more sensitive between the sexes.[23] Each animal, at the commencement of its dosing, is about 8 weeks old and its weight is fall in an interval within ±20% of the mean weight of any previously dosed mice. Groups of 20 mice were dosed by oral gavage in a stepwise procedure, using the fixed doses of 5, 50, 300 and 2000 mg/kg. The initial dose level was selected on the basis of a sighting study, as the dose was expected to produce some signs of toxicity without causing severe toxic effects or mortality. Clinical signs and conditions associated with pain, suffering, and impending death, are described in detail in a separate OECD Guidance Document. [22] Further groups of mice may be dosed at higher or lower fixed doses, depending on the presence or absence of toxicity or mortality. This procedure continued until either the dose that causes evident toxicity or more than one death was identified, or when no effects are seen at the highest dose or when deaths occur at the lowest dose.

### Statistical Analyses

Each experiment was performed at least three times and results are presented as the mean ± SD. IC50 was also calculated. Values of P<0.05 were considered statistically significant. The comparison of quantitative variables was performed using analysis of variance (ANOVA), and the differences were calculated using Tukey's test (p<0.05).

### Ethics Statement

In this study, no specific permits were required for the described field studies. The study is not privately-owned or protected in any way. The field studies did not involve endangered or protected species. All animal work was performed according to the international animal welfare guidelines, and protocols were approved by Shantou University Medical College Institutional Animal Care and Use Committee (permit numbers: SUMC2009-010). All animals used in this work were conventionally housed in facilities and were provided food and water *ad libitum*. Animal studies including infection, intraperitoneal injection, orbital venous bleeding of mice were conducted in accordance with the recommendations in the Guidelines for the Care and Use of Laboratory Animals of the Ministry of Science and Technology of the People's Republic of China ([2006]398).

## Results and Discussion

### Yields of ethanol extracts and solvent-partitioned fractions

Due to the complicated constituents and pharmacological diversity of seaweed materials, in vitro bioassay-guided fractionation has been applied to screen for biological activities.[24] We applied the same approach to identify active components for GL, initially partitioning ethanol extract constituents into solvent-partitioned fractions (Table 1).The percentage of GL weight extracted by ethanol was 14.8%. This yield was higher than yields found previously for the 70% ethanol extracts from the brown seaweed S.pallidum (12.7%), [7] but significantly lower than yields reported for ethanol extracts of seaweeds including Laminaria ochroleuca (21.2%), Saccorhiza polyschides (34.6%) and Fucus vesiculosus (24.1%). [25] These considerable differences in the yield of ethanol extract from various seaweeds might be due to species-specific differences as well as changes in extraction conditions, such as fluctuations in solvent, temperature, and duration of extraction. Among the solvent-partitioned fractions, the PE fraction (53.5) had the highest yield, followed by the AQ (20.1%), BuOH (12.1%), and EA (8.2%) fractions.

**Table 1:** Yield of ethanol extracts and solvent-partitioned fractions from GL.

Samples	Yield (%)
EE	14.8
PE	53.5
EA	8.2
BuOH	12.1
AQ	20.1

The yield of the ethanol fraction was in wt/wt of dried seaweed, and yield of the solvent-partitioned fractions was in percentage of total ethanol extract.

### Antioxidant capacities of extracts and fraction

Different antioxidant compounds may act through different mechanisms, so no single method can fully evaluate the total antioxidant capacity which depends on the method adopted and the lipid system used as substrate. [26] Therefore, in this study, the antioxidant activities of these extracts were evaluated through the use of three different in vitro methods: the DPPH radical-scavenging, deoxyribose scavenging, and β-carotene bleaching assay.

#### DPPH radical-scavenging activity

**Figure 1:**
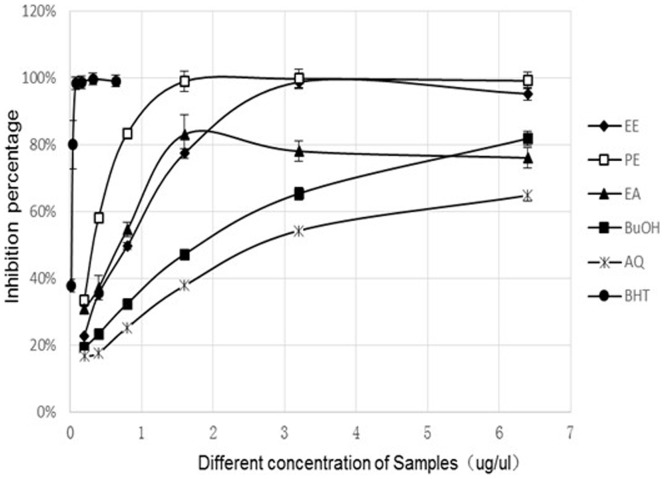
DPPH radical-scavenging activity of GL extracts.

The DPPH radical-scavenging assay has been widely accepted as a tool for estimating the radical-scavenging activity of antioxidants. Such radical scavenging is attributed to hydrogen-donating ability of the antioxidant. DPPH radical-scavenging activities of the ethanol extract and solvent-partitioned fractions are show in Fig 1.

Fig 1. shows that the PE fraction possesses the strongest DPPH-scavenging activity among different GL extracts. From Table 2, BHT, as the positive control, exhibited the highest DPPH radical-scavenging activity with the lowest IC50 (0.038 mg/ml), followed by the PE fraction (0.483 mg/ml), EE (0.701 mg/ml) and EA fraction (0.683 mg/ml). In addition, the scavenging effects increased with the increase in sample concentration.

**Table 2:** IC50 (mg/ml) of different GL extracts in all three antioxidant activity assays.

Extracts	DPPH	Deoxyribose	BCB
EE	0.701±0.019^c^	0.696±0.011^b^	0.636±0.035^c^
PE	0.483±0.015^d^	0.394±0.009^c^	0.370±0.016^d^
EA	0.683±0.017^c^	0.769±0.045^a^	0.898±0.157^b^
BuOH	1.258±0.050^b^	0.807±0.051^a^	3.043±0.294^a^
AQ	2.629±0.068^a^	0.781±0.035^a^	3.608±0.250^a^
BHT*	0.038±0.005^e^	0.006±0.001^d^	0.007±0.001^e^

* BHT was used as a standard antioxidant in all three antioxidant assays.

a,b,c,d,e Different letters in the same column indicates significant differences by Tukey's test at p<0.05.

#### Deoxyribose scavenging activity

**Figure 2:**
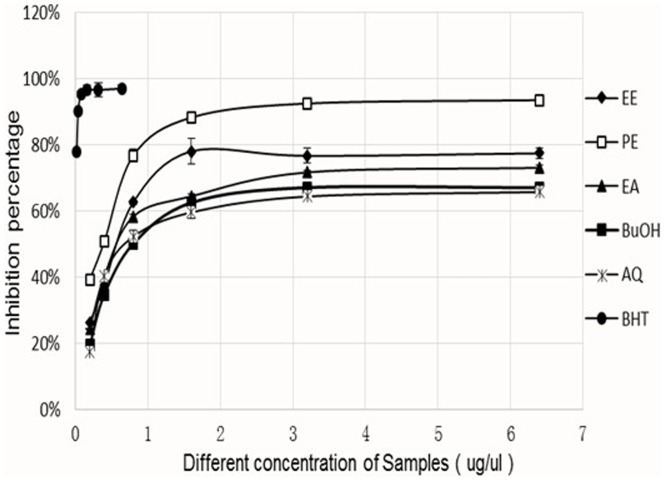
Deoxyribose scavenging activity of the GL extracts.

The important aspect of the deoxyribose assay is that it involves scavenging the hydroxyl radical, the most active reactive oxygen species.[27] The effect of extracts in scavenging OH radicals to prevent oxidative degradation of deoxyribose was determined (Fig 2).

The PE fraction (IC_50_ = 0.394 mg/ml), had the highest deoxyribose scavenging activity, with EE (0.696 mg/ml) and the EA fraction (0.769 mg/ml) following. The deoxyribose scavenging activity of each extract increased linearly with increased concentration up to approximately 1 mg/ml.

#### BCB assay

**Figure 3:**
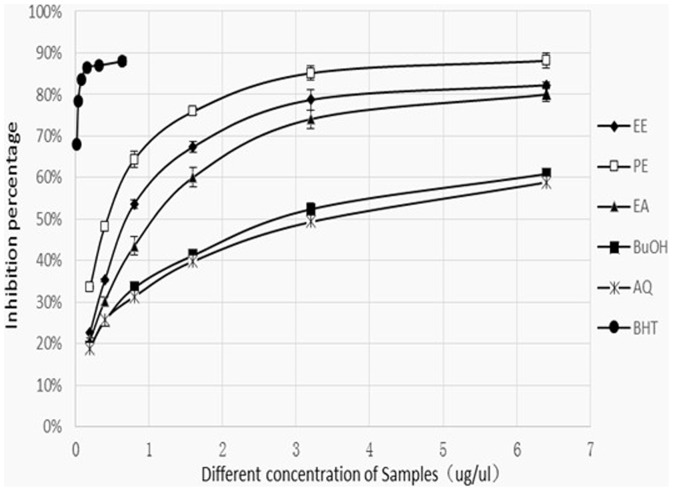
Antioxidant activity of GL extracts determined by BCB assay.

In the BCB assay, oxidation of linoleic acid generates peroxyl free radicals due to the abstraction of hydrogen atoms from the diallylic methylene groups of linoleic acid.[28] The free radical can then oxidize the highly unsaturated β-carotene. The presence of antioxidants in the extract will minimize the oxidation of β-carotene by hydroperoxides. Hydroperoxides formed in this system will be neutralized by the antioxidants from the extracts.[29] The antioxidant activity of GL extracts and fractions in the BCB assay is shown in Fig 3.

As shown in Fig 3, BHT shows the highest antioxidant activity. From Table 2, the effect of PE fraction (0.3702 mg/ml) on the coupled oxidation of linoleic acid and β-carotene was higher than other fractions.

These results show that compounds with the strongest antioxidant capacities in GL may be more soluble in a slightly less polar solvent, such as petroleum ether and ethyl acetate. Many reports have been published with the similar trends, such as for both green and brown seaweeds[7].

### Antimicrobial susceptibility testing

The results of antimicrobial screening are presented in Table 3. MIC determination indicated the GL extracts inhibited the microorganisms tested. The PE fraction exhibited broad-spectrum antimicrobial activity, even on drug-resistant Escherichia coli. The solvent used as the negative control in this test did not indicate activity on any of the microorganisms tested. Oxacillin, ampicillin, ceftazidime were also used as positive controls.

**Table 3:** Antibacterial activity of GL extracts.

strain	MIC in mg/mL								
	EE	PE	EA	BuOH	AQ	OX	AM	CE	solvent
*S. aureus* ATCC 25923	>8	2	>8	>8	>8	-	NT	NT	+
*E. coli* ATCC 25922	2	1	>8	>8	>8	NT	-	NT	+
*P.aeruginosa* ATCC 27853	>8	4	>8	>8	>8	NT	NT	-	+
*S. aureus* 2500^1^	>8	>8	>8	>8	>8	NT	+	+	+
*E. coli* 3529 1	2	2	>8	>8	>8	NT	+	+	+
*P. aeruginosa* 2470^1^	>8	>8	>8	>8	>8	NT	+	-	+

*S. aureus: Staphyloccocus aureus; E. coli: Escherichia coli; P.aeruginosa: Pseudomonas aeruginosa*

OX: Oxacillin, AM: Ampicillin, CE: Ceftazidime.

1 : *S aureus* 2500, *E coli* 3529 and *P aeruginosa* 2470 were isolated from clinic.

- indicates microorganism did not demonstrate visible growth, + inhibition the contrary, >8 No inhibition at the maximum concentration (8 mg/ml) used, NT: not tested.

### Activity against Schistosoma japonicum adult worms in vitro

Due to low fatality rates, deleterious effects on the environment and the high cost of synthetic antischistosomal drugs, scientists focus their attention on plant antischistosomal compounds, which are less hazardous to the environment than synthetic drugs.[30] Several in vitro studies have been performed to search for new active compounds against Schistosoma species [19], [31].

In this study, in vitro effects of different concentrations of the GL extracts on adult S. *japonicum* worms were evaluated. As shown in Table 4, 30 ug/ml praziquantel (PZQ), our positive control, resulted in death of the parasites within 24 h, whereas no mortality was observed in worms belonging to the negative control groups (RPMI 1640 medium and DMSO 1% plus RPMI 1640 medium). At 100 ug/ml, all the extracts exhibited significant activity by completely immobilizing all adult worms within 72 h. The PE fraction at 25 µg/mL caused the death of 40.95% of S. *japonicum* adult worms after 24 h of incubation which was higher than the EE extract, and incubation with the PE fraction at 100 µg/mL, resulted in the death of all the S. *japonicum* adult forms after 24 h.

**Table 4:** Effect of different fractions against aduit Schistosoma japonicum worms in vitro.

Group	No.^c^	Number of worms dead after incubation (100%)		
		24 h	48 h	72 h
EE				
25 ug/ml	11	0	22.75^1^	27.25^1^
50 ug/ml	11	36.35^1^	40.9^1^	71.45^1^
100 ug/ml	9	100^1,2^	100^1,2^	100^1,2^
PE				
25 ug/ml	9	40.95^1^	63.9^1^	81.25^1^
50 ug/ml	11	82.95^1,2^	87.5^1,2^	100^1,2^
100 ug/ml	8	100^1,2^	100^1,2^	100^1,2^
1640^a^	8	0	0	0
1%DMSO^a^	8	0	0	0
PZQ^b^	9	11.1	44.4^1^	66.7^1^

a RPMI 1640 medium and 1% DMSO in RPMI 1640 medium were used as negative control groups.

b Praziquantel (PQZ, 30 ug/ml) was used as positive control groups. Data are presented as the mean of four experiments.

c Number of worms tested.

1 Drug group compared with RPMI 1640, 1%DMSO negative control group, both P values<0.05;

2 Drug group compared with PZQ positive group, both P values<0.05;

### Gas chromatography-mass spectrometry (GC-MS) analysis of the PE fraction

**Figure 4:**
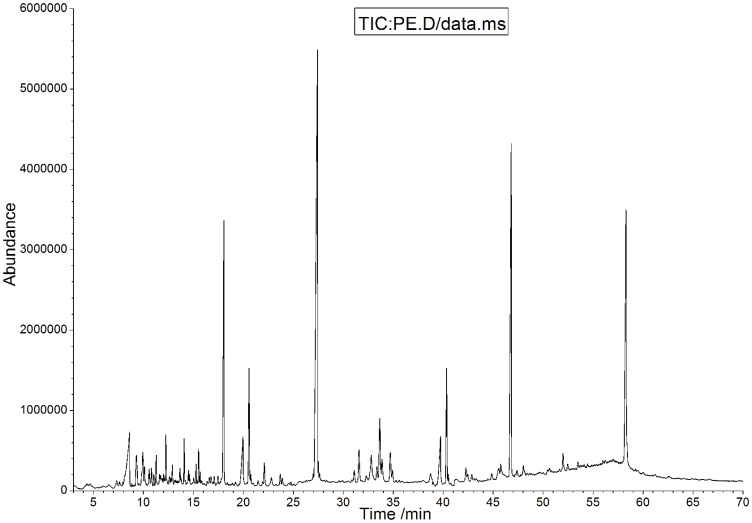
The total ion chromatogram of volatile constituents in the PE fraction.

The previous results show that the PE fraction has the highest antioxidant and antibacterial activities among all the GL extracts, consistent with the yield of PE fraction being much higher than other fractions. Therefore, we analyzed the chemical composition of the PE fraction in order to investigate the relationships between bioactivity and chemical composition. The compounds in the PE fraction were separated and determined using GC-MS analysis. The total ion chromatogram (TIC) of the volatile constituents from the PE fraction is shown in Figure 4. The percentage composition of the PE fraction was computed by the normalization method from the GC peak areas, without using correction factors. Identification of the constituents was based on comparison of their Kovats Index (KI) and MS fragmentation pattern with reference compounds in the NIST Mass Spectral Search Program database.

GC-MS analysis of the PE fraction resulted in identification of 25 chemical compounds, with greater than 90% similarity with standard mass spectra, representing 75.17% of the relative area in the PE fraction (Table 5). This fraction is characterized by the presence of organic acid ester (26.34%), fatty acids (including 20.68% palmitic acid and 2.27% tetradecanoic acid), sterol (including 9.16% cholesterol),and amide compounds (6.50%).

**Table 5:** Chemical composition of the PE fraction identified by GC-MS and Kovats Index.

NO.	RT a	Constituents	RI^b^	%^c^
1	8.628	Ethyl hydrogen succinate	1096.4	3.95
2	8.649	Benzoic acid	1196.9	0.07
3	12.009	1-Hexadecene	1390.5	0.22
4	14.085	2,4-Di-tert-butylphenol	1511.4	0.79
5	14.514	Dihydroactinidiolide	1535.2	0.32
6	18.058	Heptadecane	1701.4	6.68
7	19.971	Tetradecanoic acid	1772.4	2.27
8	22.105	2-Pentadecanone, 6,10,14-trimethyl	1844.1	0.65
9	23.693	Ethyl pentadecanoate	1894.5	0.27
10	26.000	Butyl isobutyl phthalate	1960.8	0.42
11	27.420	n-Hexadecanoic acid	1992.3	20.68
12	27.515	Eicosane	2000.8	0.26
13	31.095	Docosane	2200.0	0.37
14	31.582	Phytol	2212.4	1.13
15	32.798	Heptadecyl acetate	2244.3	1.24
16	33.370	Linoleic acid ethyl ester	2259.3	0.48
17	33.653	Ethyl Oleate	2266.7	2.26
18	34.921	Tricosane	2300.0	0.4
19	39.471	Ethyl arachidonate	2419.5	4.27
20	39.760	Methyl eicosapentaenoate	2427.3	6.98
21	42.281	Cyclotetracosane	2495.2	0.26
22	46.815	Mono-(2-ethylhexyl)phthalate	2748.5	11.08
23	48.026	1-Hexacosene	2876.7	0.39
24	51.995	Cis-9-Octadecenoamide	2973.4	0.57
25	58.290	Cholesterol	3403.2	9.16

a Retention times (minute).

b Retention index was calculated from our analyses with respect to a series of n-alkenes.

c Percentage of extracted amount to total.

Some of the identified compounds in the PE fraction have been reported to exhibit antioxidant and antimicrobial activities. Palmitic acid, as the most abundant component of the PE fraction, has been reported to have antioxidant, hypocholesterolmic nematicide, pesticide, antiandrogenic flavor, hemolytic and 5-alpha reductase inhibitor activity. [32] Tetradecanoic acid, phytol and ethyl arachidonate were also identified to have high antioxidant, antimicrobial and anticancer activity,[33] and 2,4-di-tert-butylphenol has been reported to have antioxidant effects.[34] Cholesterol was the principal sterol in GL. The health benefits of sterols from photosynthetic organisms include anti-inflammatory activity,[35] protection against oxygen free radicals,[36] and also prevention of β-amyloid-induced neurotoxicity.[37] This link between phytosterols and ROS scavenging capacity,[36] may help to explain the high antioxidant activity of the PE fraction. Interestingly, some of these compounds, such as dihydroactinidiolide, are used as pheromones by some higher plants and insects.

Some GL compounds in our GC-MS analysis, have known antibacterial activity. A number of free fatty acids, as well as their esters, are known to possess antibacterial activity against Gram-positive bacteria, [38], [39]and other synthetic fatty acid analogs of cholesterol show excellent antibacterial activity *in vitro*.[40] Compounds comprising a small fraction of the extract, such as palmitic acid, have been reported to have significant antimicrobial activity. [41]

The components collectively present at low concentrations in our extract might be involved in some type of synergism with the other active compounds. MacDonald and Fahien found that initiation of insulin release by succinate esters, through mitochondrial metabolisms, is sufficient to initiate and support insulin release from βcells. [42], [43] Benzoic acid is used as an antifungal agent and food preservative. Phytol has also been identified to have strong antioxidant, antimicrobial and anticancer activity,[33] and butyl-isobutyl-phthalate is a potential α-glucosidase inhibitor for type II diabetes treatment.[44]

The PE fraction, as a mixture, exhibits strong activity against the adult form of the human pathogenic trematode S. *japonicum* worms. However, none of the compounds, identified by GC-MS, have been reported to have activity against Schistosoma. This suggests that the in vitro schistosomicidal activity of the PE fraction may be either related to minor constituents present in the extract or governed by more intricate synergistic or additive relationships.

### Cytotoxicity of fractions

**Figure 5:**
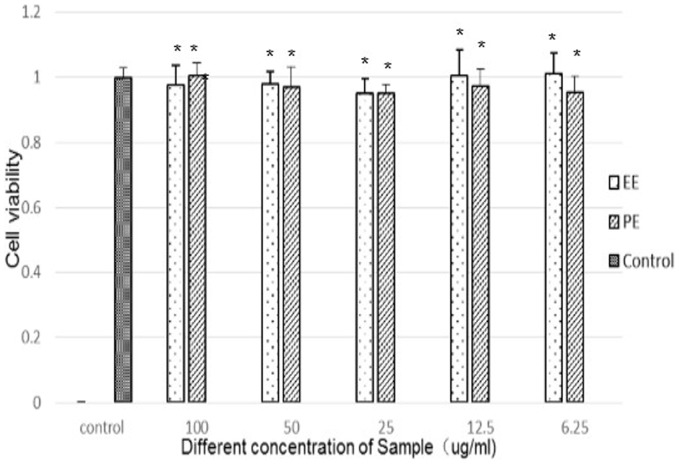
Cytotoxic effect of extracts on RAW264.7 murine macrophage cells. After the cells were incubated with the EE and PE fraction (0, 6.25, 12.5, 25, 50, 75, and 100 ug/mL) for 24 h, the viability was measured by MTT assay. * Drug group compared with control group, both P values >0.05;

Because the EE and PE fractions had the highest antioxidant and antibacterial activities compared to the other GL extracts, an MTT assay was performed to determine the cytotoxic effects using the RAW264.7 murine macrophage cell line. As shown in Fig 5, exposure of cells to different concentrations of the EE and PE fractions (0, 6.25, 12.5, 25, 50, and 100 µg/mL) did not cause any cytotoxicity at concentrations up to 100 ug/ml.

#### Acute oral toxicity in mice

Mice were administered various doses of extract to determine in vivo toxicity. No mortality or severe toxic effects were seen even at the highest dose of 2000 mg/kg (Table 6). Hence, based on the OECD criteria, the *GL* extracts and their fractions are of relatively low acute toxicity hazard and their expected LD50 values exceeded 2000 mg/kg without the need for testing.

**Table 6:** Acute oral toxicity in mice-fixed dose procedure.

Dose(mg/kg)	Result of the study				
	TE	PE	EA	BuOH	AQ
5	+	+	+	+	+
50	+	+	+	+	+
300	+	+	+	+	+
2000	+	+	+	+	+

+ ineicates a 100% survival rate, no severe toxic effect, clinical signs and conditions associated with pain, suffering, or impending death. Detailed described are in a separate OECD Guidance Document.[22]

## Conclusions

In the present work, it was the first report that GL extracts possess antioxidant, antibacterial and antischistosomal activities. The PE fraction has the most antioxidant, antibacterial and antischistosomal activities. The chemical composition of 75.17% of the PE fraction was identified and analyzed. Results from analytical experiments and limited biological assessments indicate that the predominance of biological activity in GL extracts could be due to the identified chemical composition. Simultaneously, the PE fraction does not display toxicity in either in vitro cytotoxicity or in vivo acute oral toxicity assays. The results of the present work indicate that the PE fraction of GL could be used as a source of natural antioxidants, antimicrobial, and schistosomicidal agents in food preservation and human health.
